# 引言

**DOI:** 10.3724/SP.J.1123.2021.08030

**Published:** 2021-09-08

值此恩师张玉奎院士八十华诞之际,我们策划出版了《色谱》2021年9月和10月的两期专辑,以表达对先生的由衷敬意和深深祝福。

张玉奎院士作为我国色谱领域的先驱者,不仅极大地推动了色谱学科的纵深发展,而且还注重完成国家任务,加快了国产色谱仪的产业化进程。七八十年代,先生在色谱热力学和动力学研究方面做出了突出的贡献,促进了液相色谱专家系统的建立和完善;九十年代,先生致力于环境污染物、天然产物、临床小分子等复杂体系的分离分析,将色谱用于解决生命健康密切相关领域的实际问题;二十一世纪以来,先生高瞻远瞩,瞄准分析化学和生命科学的前沿,面向国家重大需求,投身于生物大分子的高效分离与表征研究,在蛋白质组定性定量、结构和相互作用新技术新方法方面取得了显著进展,为解决精准医学、环境毒理、合成生物学等领域中的瓶颈问题提供了关键的技术支撑。

先生不仅在色谱领域取得了卓越的学术成就,而且言传身教,培养了一大批杰出的中青年学术带头人和优秀的企业研发骨干。同时,先生还为《色谱》的发展倾入了大量的心血,使之成为在该领域各行各业备受瞩目的期刊。

本专辑采编了反映当前色谱最新前沿领域的文章。在此,衷心感谢每位作者为本专辑撰写的最新力作;衷心感谢每位审稿专家为稿件提出的真知灼见。

谨以此专辑恭贺张玉奎院士八十华诞。祝先生福如东海,寿比南山,身体健康,诸事顺遂!

中国科学院大连化学物理研究所

本专辑客座主编

张丽华 研究员


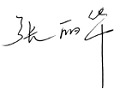


2021年8月31日

